# Experimental Study on Shear Rheological Polishing of Si Surface of 4H-SiC Wafer

**DOI:** 10.3390/mi14040853

**Published:** 2023-04-14

**Authors:** Peng Li, Julong Yuan, Minghui Zhu, Jianxing Zhou, Binghai Lyu

**Affiliations:** 1 College of Mechanical Engineering, Zhejiang University of Technology, Hangzhou 310014, China; 2 Key Laboratory of Special Purpose Equipment and Advanced Processing Technology, Ministry of Education and Zhejiang Province, Zhejiang University of Technology, Hangzhou 310014, China

**Keywords:** shear rheological polishing, 4H-SiC wafer, Si surface, Taguchi method, material removal rate, surface roughness

## Abstract

In this study, shear rheological polishing was used to polish the Si surface of six-inch 4H-SiC wafers to improve polishing efficiency. The surface roughness of the Si surface was the main evaluation index, and the material removal rate was the secondary evaluation index. An experiment was designed using the Taguchi method to analyze the effects of four critical parameters (abrasive particle size, abrasive particle concentration, polishing speed, and polishing pressure) on the Si surface polishing of SiC wafers. By evaluating the experimental results for the signal-to-noise ratio, the weight of each factor was calculated using the analysis of variance method. The optimal combination of the process parameters was obtained. Below are the weightings for the influence of each process on the polishing result. A higher value for the percentage means that the process has a greater influence on the polishing result. The wear particle size (85.98%) had the most significant influence on the surface roughness, followed by the polishing pressure (9.45%) and abrasive concentration (3.25%). The polishing speed had the least significant effect on the surface roughness (1.32%). Polishing was conducted under optimized process conditions of a 1.5 μm abrasive particle size, 3% abrasive particle concentration, 80 r/min polishing speed, and 20 kg polishing pressure. After polishing for 60 min, the surface roughness, R_a_, decreased from 114.8 to 0.9 nm, with a change rate of 99.2%. After further polishing for 60 min, an ultrasmooth surface with an R_a_ of 0.5 nm and MRR of 20.83 nm/min was obtained. Machining the Si surface of 4H-SiC wafers under optimal polishing conditions can effectively remove scratches on the Si surface of 4H-SiC wafers and improve the surface quality.

## 1. Introduction

Power electronics technology has been advancing toward increased efficiency, power density, and integration system [1,2]. Traditional Si-based semiconductor materials have encountered bottlenecks in the development of modern power devices [3]. SiC has replaced Si because of its high hardness, chemical inertia, wide bandgap, and electrical stability at high temperatures [4,5]. SiC has become a potential material for microelectromechanical systems (MEMS) and power devices in extreme environments [6,7]. The processing quality of single-crystal SiC substrates significantly influences the device’s performance. The quality of the Si surface directly influences the quality of the epitaxial thin films and device performance [8]. However, the efficient and high-quality polishing of SiC substrates remains challenging because of their high hardness and chemical stability [3]. Therefore, there is an urgent need to develop efficient and high-quality surface-polishing methods for SiC wafers.

SiC substrates are mainly polished using chemical mechanical polishing (CMP), magnetorheological polishing (MRF), or electrochemical mechanical polishing (ECMP). Wang et al. [9] used photocatalysis to enhance the polishing effect of CMP and processed the surface of a 4H-SiC substrate. The material removal rate (MRR) reached 694 nm/h, and the surface roughness (R_a_) was 0.489 nm. Deng et al. [10] used CMP based on the electron-Fenton reaction to process single-crystal 6H-SiC. After polishing, the surface roughness R_a_ was 31 nm, and the MRR was 443 nm/h. The chemical action of a CMP polishing solution is enhanced by adding a catalyst. The polishing efficiency and surface quality also improve. In addition, it is challenging to balance the chemical action and mechanical removal.

Pan et al. [11] used MRF to process single-crystal 6H-SiC, achieving an R_a_ value of 1.9 nm. Yin et al. [12] applied electromagnetic field-excited high-polishing mode magnetorheological polishing of single-crystal SiC and obtained a smooth wafer surface with an R_a_ value of 0.6 nm. MRF can be successfully applied to the high-quality processing of hard and brittle materials. However, this method is complex, inconvenient to operate, and involves the high cost of the magnetorheological fluid.

Wang et al. [13] conducted polishing experiments using optimized test parameters. The results showed that a machined surface with a 0.408 nm surface roughness of SiC could be obtained during the precision polishing stage. Ballarin et al. [14] used an HF solution as an electrolyte to polish the surface of polycrystalline SiC during ECMP. Finally, a surface with an RMS of 8.3 nm was obtained; however, the MRR was still low. Moreover, if the concentration of the HF solution is very low or very high, it will increase the R_a_ value. ECMP can yield decreased surface roughness and improved surface morphology. However, the MRR is low, and the removal uniformity is poor; hence, it is impossible to achieve efficient and high-quality surface processing.

However, these polishing methods can provide good surface roughness. Problems such as low polishing efficiency, complex equipment, and complicated processes exist. Therefore, it is necessary to develop a method for polishing SiC wafers with low cost, minimal damage, and high efficiency.

Shear rheological polishing (SRP) combines the shear thickening effect of non-Newtonian fluids with wear particles to form a “flexible fixed abrasive tool” to quickly and uniformly remove the surface material of a workpiece, which can achieve low damage and high-efficiency flexible polishing of the specially shaped workpiece surface [15,16]. Shao et al. [17], using the optimized processing parameters of the SRP method, found that the surface roughness of quartz glass decreased from 120 nm to 2.1 nm MRR to 126.2 nm/min after polishing for 8 min. Yang et al. [18] polished a cylindrical roller by adjusting SRP process parameters. After polishing for 90 min under the optimum conditions, the R_a_ values of the rolling surface and end face of the cylindrical roller decreased from approximately 130 nm to 9.8 and 9.4 nm, respectively. Duan et al. [19] investigated the effects of various single and mixed wear particles on K9 glass processing using the SRP method. After polishing the K9 glass for 35 min under optimized abrasive conditions, the original roughness of the K9 glass surface (230.7 nm) decreased to 1.43 nm. Yuan et al. [20] applied chemical-assisted rheological polishing of alloy turbine blades and found that the surface roughness, R_a_, decreased from 72.3 to 4.2 nm in 9 min. Ke et al. [21] machined the rake face of a cemented carbide blade under optimized SRP process conditions. After polishing for 15 min, the surface roughness of the chip-breaking groove decreased from 120 to 7.2 nm.

The shear rheological polishing method was adopted in this study to achieve efficient and high-quality polishing of SiC wafers. The shear thickening effect of the shear rheological polishing fluid was used to achieve the flexible removal of rough peaks on the wafer surface. The aim was to simultaneously improve the surface quality of SiC wafers and achieve efficient machining. An orthogonal experiment was designed using the Taguchi method. The effects of abrasive-particle size, abrasive-particle concentration, polishing speed, and polishing pressure on R_a_ and MRR were analyzed. The process parameters were then optimized.

## 2. Materials and Methods

### 2.1. Principle of Shear Rheological Polishing of SiC Wafer

**Figure 1:**
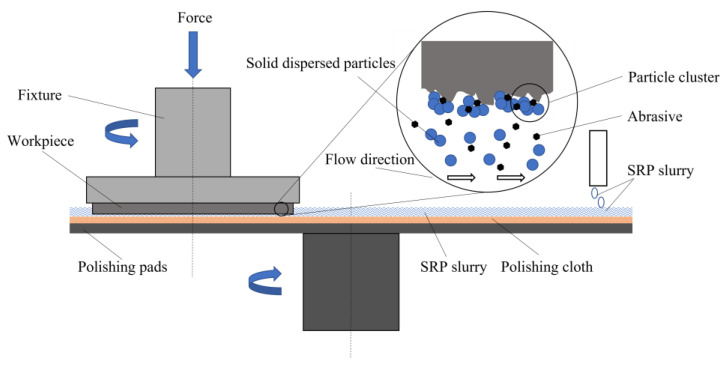
Schematic of SRP of SiC wafer.

During the shear rheological polishing of a SiC wafer, the wafer is subjected to a specific pressure and produces rapid relative motion with the polishing fluid (Figure 1). Shear thickening occurs when the machined surface of the wafer and the polishing liquid are subjected to high shear strain. The polymer particles in the polishing solution combine with the wear particles to form a “particle cluster,” producing a “flexible fixed abrasive tool” for fitting the wafer surface. The wafer surface material can be removed rapidly [22]. This is because the shear stress at the rough peak of the wafer surface is higher than that at the lower plane of the wafer. The relative removal efficiency of the wafer microrough peak is higher and achieves rapid planarization of the SiC wafer surface, thus, realizing wafer processing with high efficiency and minimal damage. This method achieves higher polishing efficiency than the CMP method presented previously. The method achieves improved polishing efficiency and post-polishing accuracy compared to the ECMP and MRF methods presented previously.

### 2.2. Experimental Process and Conditions

**Figure 2:**
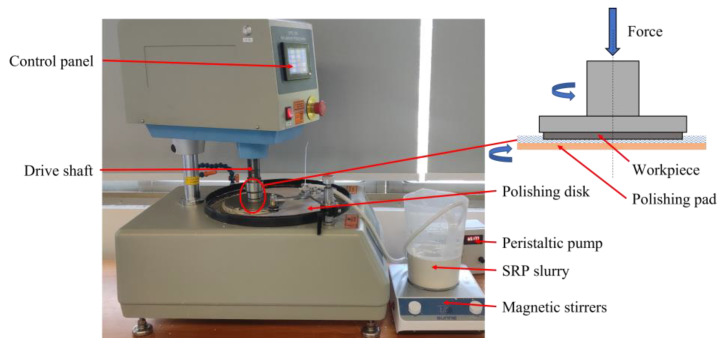
SRP experimental setup and its schematic.

Figure 2 shows the experimental setup of the SiC wafer SRP device. The wafer was clamped to the drive shaft fixture, rotated, and pressed. A polishing pad was placed on the polishing disc. During the polishing process, the polishing disc rotated the polishing fluid. The polishing pad increased the adsorption force of the polishing disk on the polishing liquid. Consequently, the speed loss of the polishing liquid caused by its fluidity decreased. Increasing the relative shear rate of the contact area enhanced the shear-thickening effect of the polishing fluid. The polishing pressure applied by the driving shaft increased the bonding effect of the polishing solution on the wafer surface. This ensured that the “particle cluster” wrapped with wear particles effectively contacted the wafer. A cutting force was generated on the wafer surface to achieve material removal. The diameter of the 4H-SiC wafer used in this study was 150 mm. The material properties are listed in Table 1. Table 2 lists the specific experimental conditions for SRP. The rotational speed of the polishing disk was measured as the polishing speed.

**Figure 3:**
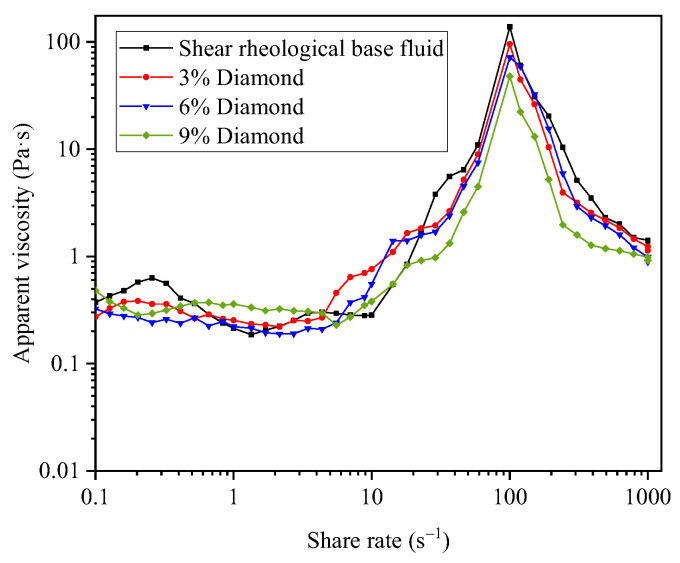
Rheology curves.

The SRP polishing fluid was prepared as follows. First, deionized water and abrasive particles were uniformly mixed to obtain a conventional water-based polishing fluid; Next, the polymer particles were added and evenly mixed to form a shear rheological polishing fluid. The Morse hardness of the 4H-SiC wafer was as high as 9.2, indicating that it is a typically hard and brittle material. Therefore, diamond particles were used as the abrasive shear rheological polishing fluid. Diamond with average particle sizes of 0.5, 1, and 1.5 μm was used for polishing with different concentrations of shear rheological polishing fluid to investigate the effects of the abrasive particle size and concentration on the polishing effect. The rheological behavior curves of the shear rheological polishing fluid plotted for different concentrations of wear particles of the same particle size were measured using a stress-controlled rheometer (HAAKE Mars40, Thermo Fisher, Germany). With a cone plate (diameter: 25 mm, cone angle: 2°, gap: 0.1 mm), the test temperature was 25 °C. The measurement results are presented in Figure 3. Figure 3 shows the rheological properties of a shear rheological base fluid and three SRP polishing fluids with a 1.5 μm diamond grit concentration. The rheology of the polishing fluid exhibited an instantaneous phenomenon. When the shear rate exceeded approximately 10 s^−1^, the fluid viscosity increased slowly, reaching approximately 100 s^−1^ and resulting in a strong shear thickening effect. The viscosity reached its peak value. With a further increase in shear rate, the fluid viscosity decreased significantly.

**Figure 4:**
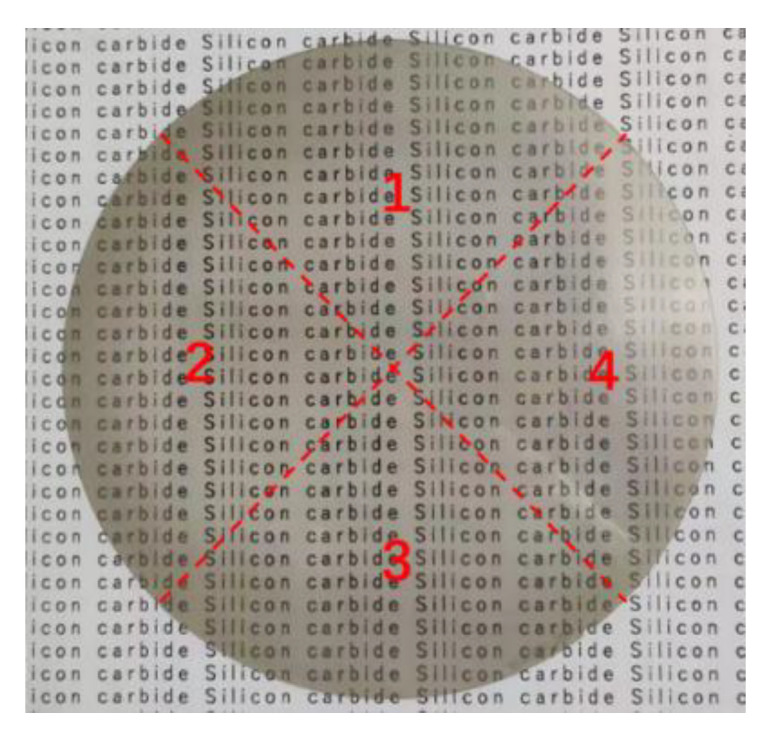
Schematic of measurement area on wafer surface.

The surface roughness and surface microtopography of the four different wafer regions were measured using an optical three-dimensional surface profiler (Super View W1, CHOTEST, China). The measurement area is illustrated in Figure 4. The text markings in the background of Figure 4 are to reflect the light transmission of the silicon carbide wafer. The sampling area was 0.5 mm × 0.5 mm. This device had a measurement error of 0.1 nm. The average values of the six different locations in each region were adopted as the values of the region owing to the large sample area. The final experimental results were the average values of the four regions. Before and after polishing, the wafer quality was measured three times and averaged using a precision balance (MSA225S−CE, Sartorius, Germany, accuracy of 0.01 mg). The surface morphology of the wafer was observed using the ultradepth of a field microscope (VHX−7000, Keyence, Japan) and a scanning electron microscope (Sigma−300, Zeiss, Germany).

### 2.3. Experimental Design

The abrasive particle size, abrasive particle concentration, polishing speed, and polishing pressure were the main process parameters influencing the R_a_ value and MRR of the SiC wafer. In this study, an L9 (3^4^) orthogonal experiment with three levels and four factors was designed using the Taguchi method [23], as listed in Table 3. Average response analysis was performed using the calculated average response value. The optimal level for each factor was determined.

In the experiment, the best combination of the four groups of parameters was selected by analyzing the signal-to-noise ratio (*S*/*N*) [24]. When adopting R_a_ as the standard, Equation (1) is used to perform the parameter design for the expected small characteristic. By considering the MRR as the standard, Equation (2) is used to perform the parameter design of the expected large characteristic.

(1) $$S/N=-10\mathrm{lg}(\frac{1}{n}\sum_{j=1}^{n}X_{ij}^{2})$$

(2) $$S/N=-10\mathrm{lg}(\frac{1}{n}\sum_{j=1}^{n}\frac{1}{Y_{ij}^{2}})$$

where *n* is the number of surface roughness test areas (its value is 4), *i* is the experimental serial number, and *X_ij_* and *Y_ij_* are the *R*_a_ and MRR values of the different measurement areas of the wafer, respectively. *X_ij_* was determined through direct measurements. MRR is expressed by Equation (3) as follows:

(3) $$Y=10^{7}\Delta m/\rho St$$

where Δ*m* is the difference between the wafer quality before and after polishing, expressed in g. *ρ* is the density, with a value of 3.12 g/cm^3^. *S* is the processing area, with a value of 176.71 cm^2^. *t* is the time measured in min.

## 3. Results and Discussion

### 3.1. Analysis of Average Response of S/N

**Figure 5:**
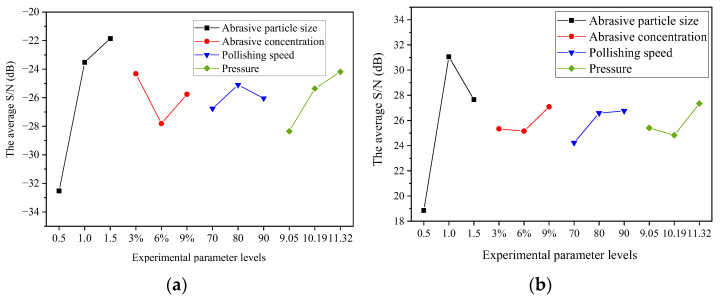
Effects of various factors on (**a**) R_a_ and (**b**) MRR.

The surface roughness of the Si surface of the 4H-SiC wafer, R_a_, and the ratio of the MRR to *S*/*N* are listed in Table 4. The ratio of R_a_ to the MRR on the Si surface of the wafer changed, as depicted in Figure 5. The X- and Y-axes in Figure 5a,b have the same interpretation. The *X*-axis, from left to right, shows the following four factors: polishing grain size, polishing concentration, polishing speed, and polishing pressure. Each factor contains three parameters. The *Y*-axis represents the average *S*/*N*. Higher *S*/*N* values indicate better polishing results.

### 3.2. Effect of Abrasive Particle Size

**Figure 6:**
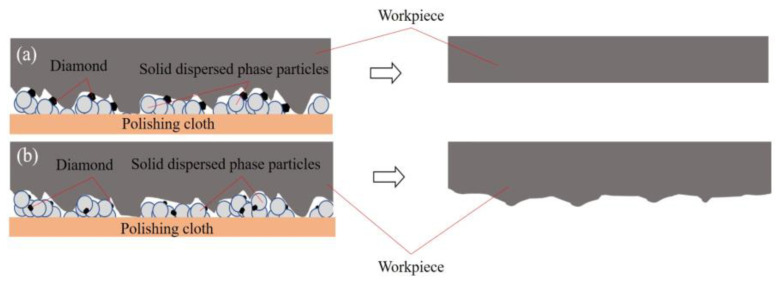
Contact models of abrasive particles with different particle sizes: (**a**) abrasive particles with large particle size. (**b**) Abrasive particles with small particle size.

As shown in Figure 5a, the average *S*/*N* value of R_a_ increased with increasing wear particle size. The increasing trend slowed when the average particle size exceeded 1.0 μm. In the “particle cluster” produced by the shear thickening effect, the degree of exposure of the abrasive cutting edge increased with increasing particle size (Figure 6). The rough peaks on the wafer surface could be effectively contacted in the same polishing process to flatten the wafer surface and decrease the surface roughness. As shown in Figure 5b, the average *S*/*N* of the MRR reached a peak value of 31.44 when the average particle size was 1.0 μm and slightly decreased when the particle size increased to 1.5 μm. In contrast, when the particle size was very small, the wear particles in the “particle cluster” were difficult to contact with the wafer surface, sharply reducing the actual processing of the wear particles and decreasing the MRR [25]. However, at the same mass fraction, the number of wear particles per unit volume of the polishing solution continued to decrease with increasing wear particle size, reducing the cutting edges and MRR involved in wafer surface material removal.

### 3.3. Effect of Abrasive Particle Concentration

With an increase in the wear particle concentration, the average *S*/*N* values of the R_a_ and MRR for the shear rheological polishing of the SiC wafers were similar. All showed a decreasing trend at first and then increased. They reached peaks of −24.32 and 27.09 at concentrations of 3% and 9%, respectively. As the concentration increased, the peak viscosity of the polishing fluid decreased (Figure 3). The adhesion to the polishing pad and the holding force of the “particle cluster” to the abrasive particles decreased. In addition, the shear force on the wafer surface decreased, reducing the polishing efficiency. Therefore, the average *S*/*N* values of the R_a_ and MRR decreased. When the concentration increased to 9%, the increase in the number of wear particles effectively involved in cutting the surface roughness peak of the wafer increased the MRR. Hence, the average *S*/*N* values of the R_a_ and MRR increased.

### 3.4. Effect of Polishing Speed

**Figure 7:**
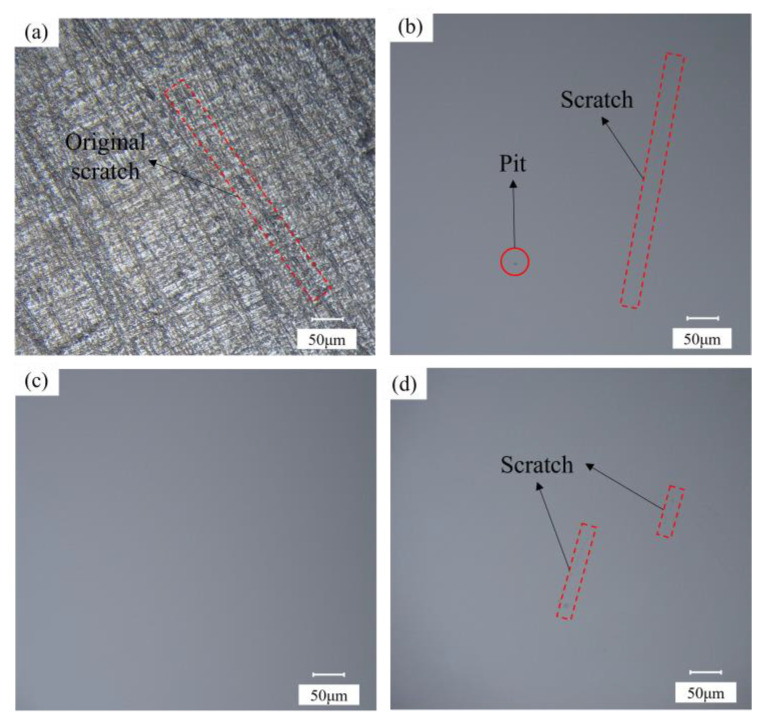
Surface profile of wafer at different polishing speeds: (**a**) original surface, (**b**) 70 r/min, (**c**) 80 r/min, and (**d**) 90 r/min.

With an increase in polishing speed, the average *S*/*N* of R_a_ initially increased and then decreased slightly. The average *S*/*N* of the MRR initially increased and then increased gradually. In contrast, the shear thickening effect of the polishing solution was directly influenced by the polishing speed. The shear rate between the wafer and polishing liquid increased with increasing polishing speed. Consequently, the holding force of the “particle cluster” on the wear particles in the contact area increased. The force acting on the surface roughness peak of the wafer increased. Moreover, the surface planarization rate increased, and R_a_ decreased rapidly. However, an increase in the relative velocity between the polishing liquid and the wafer increased the MRR. However, when the speed was increased to 90 r/min, the original damage to the wafer surface was eliminated faster, and new surface damage occurred. Figure 7 shows the surface morphology of the wafer at different polishing speeds. At low speeds, the initial damage was not eliminated. When the speed was very high, the intensity of the shear thickening effect increased sharply. The impact force of the abrasive particles on the wafer surface exceeded the brittle fracture value of the Si surface of the 4H-SiC wafer under the entrapment of the polymer particles. This caused new processing damage, increasing the R_a_ value [26].

### 3.5. Effect of Polishing Pressure

With an increase in the polishing pressure, the average *S*/*N* value of R_a_ showed a continuous upward trend. When the polishing pressure increased from 16 to 20 kg, the wear particles contacted with the small “micro-rough peak” on the wafer surface under normal force. The number of rough peaks involved in cutting increased to achieve rapid flattening of the wafer surface and reduce roughness. The average *S*/*N* of the MRR reached the lowest value (24.83 at a pressure of 18 kg). This is because an increase in pressure decreases the polishing fluid between the polishing pad and the wafer. The number of wear particles involved in effective processing and polishing efficiency decreased. When the polishing pressure was further increased, the abrasive particles could cut rougher peaks of different heights simultaneously. However, the adhesion of the polishing fluid in the contact area to the polishing pad increased. Therefore, the speed loss was smaller. The shear thickening strength and the force applied to the wear particles were enhanced. The MRR on the wafer surface increased.

### 3.6. Analysis of Variance

**Figure 8:**
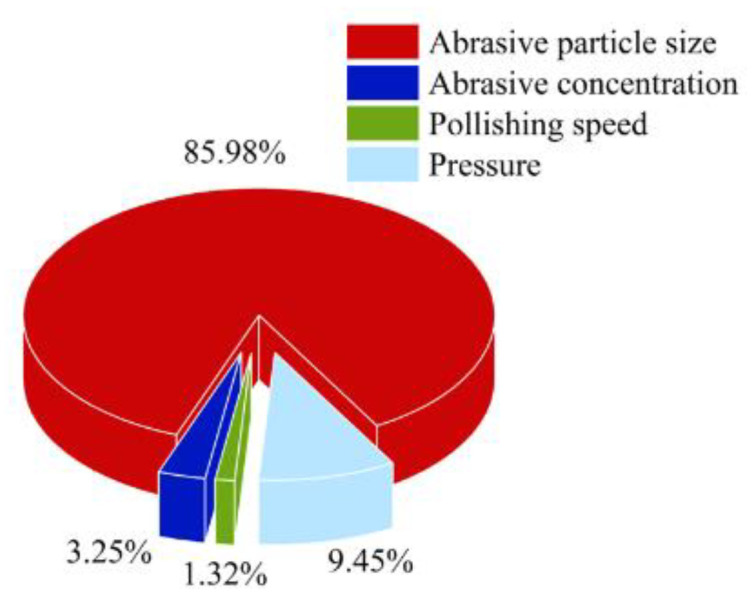
ANOVA results for R_a_.

Analysis of variance (ANOVA) was used to quantify the influence of the process parameters on R_a_ during the Si polishing of the 4H-SiC wafers. The ANOVA results for R_a_ are presented in Figure 8. The abrasive particle size (85.98%) had the most significant influence on the surface roughness, followed by the polishing pressure (9.45%) and abrasive concentration (3.25%). The polishing speed had the least significant effect on surface roughness (1.32%).

### 3.7. Verification Experiment on Shear Rheological Polishing of Si Surface of SiC Wafer

**Figure 9:**
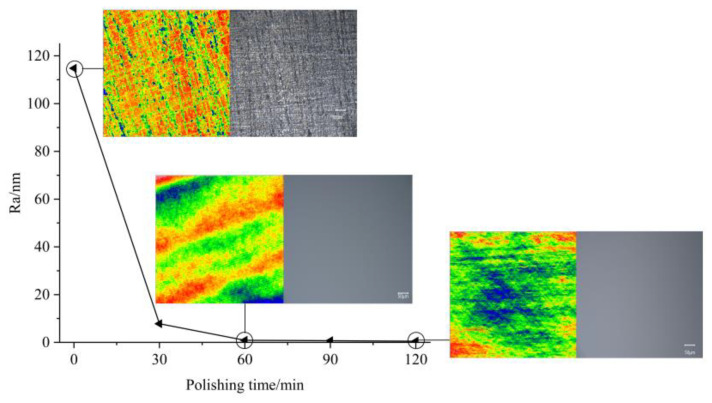
Variations in R_a_ and surface profile with polishing time.

**Figure 10:**
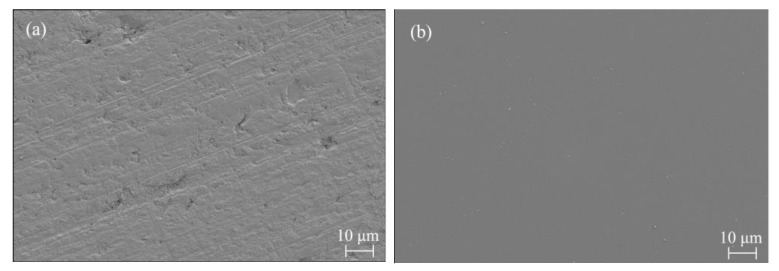
SEM micrographs of workpiece surface (**a**) before polishing and (**b**) after polishing.

**Figure 11:**
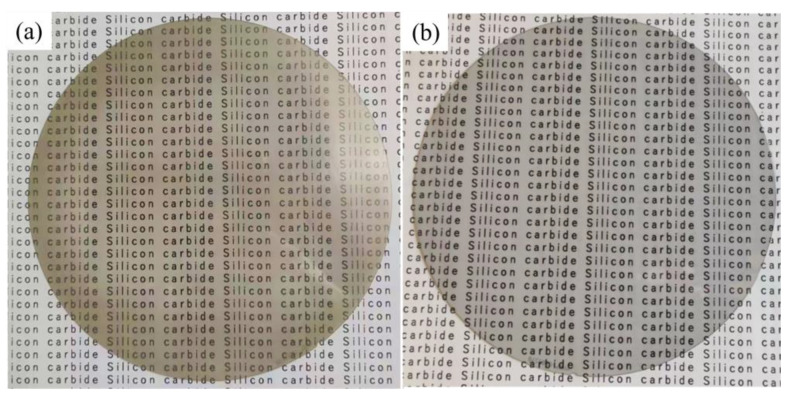
Si surface of 4H-SiC wafer before and after polishing: (**a**) original surface; (**b**) after polishing.

A horizontal response analysis of the *S*/*N* based on the Taguchi method was performed. The optimal combination of the process parameters obtained by synthesizing and recombining the optimized process parameters, R_a_ and MRR, is as follows. The particle size was 1.5 μm. The particle concentration was 3% (mass fraction). The polishing speed was set to 80 r/min, and the polishing pressure was set at 20 kg. After polishing for 60 min for this combination, the surface roughness R_a_ decreased from 114.8 to 0.9 nm at a rate of 99.2%. After further polishing for 60 min, an ultrasmooth surface with a surface roughness R_a_ of 0.5 nm and an MRR of 20.83 nm/min was obtained. The surface roughness of the wafer was not perfectly uniform in all areas after polishing, and variability was observed. Higher Ra values tended to be obtained in the center and edge areas of the wafer, with the lowest Ra values in the areas between. This is because the center of the wafer has the slowest relative velocity of the fluid to the workpiece and, therefore, has the worst material removal, which has not yet been achieved when the optimal surface quality is obtained in other areas. In contrast, the edge area is prone to overpolishing or excessive impact owing to the high relative velocity of the fluid to the workpiece, increasing the Ra value in this area. Figure 9 shows the changes in R_a_ and the surface topography of the Si plane of the 4H-SiC wafer with time during polishing. Figure 10 shows the micromorphology of the Si surface before and after polishing determined using scanning electron microscopy (SEM). The contrast before and after polishing the Si surface of the 4H-SiC wafer is shown in Figure 11. The text markings in the background of Figure 11 are to reflect the difference in light transmission of the wafer before and after polishing. In addition, the R_a_ and MRR results obtained in this experiment were highly reproducible, as all the experimental conditions were consistent. In the repeated experiments, the values of R_a_ and MRR fluctuated within a relatively narrow range.

A comparison between the above experimental results for shear rheology polishing and those of CMP shows that the MRR of CMP is generally lower than 1000 nm/h. The shear rheology polishing method can achieve 1200 nm/h, significantly shortening the polishing time. In addition, subnanometer surface roughness can be achieved. Compared to MRF, polishing solutions are less costly and do not require complex equipment control. The polishing uniformity is better than that of ECMP, improving the surface roughness.

## 4. Conclusions

In this study, the Si surface of a 4H-SiC wafer was polished using the shear rheological polishing method. Based on the Taguchi method, the effects of the abrasive particle size, abrasive particle concentration, polishing speed, and polishing pressure on the Si surface polishing of 4H-SiC wafers were investigated. The influence weight of the process parameters on R_a_ was determined using ANOVA. The best combination of the four process parameters was determined via an average *S*/*N* analysis. The following conclusions were drawn.

1. Based on the ANOVA results, the wear particle size (85.98%) had the most significant influence on the surface roughness. This was followed by the polishing pressure (9.45%) and abrasive concentration (3.25%). The polishing speed had the least significant effect on surface roughness (1.32%);
2. Through an average response analysis of the *S*/*N*, the optimal process parameters obtained by synthesizing the optimized process parameters (R_a_ and MRR) and recombining them were as follows. The particle size was 1.5 μm, the particle concentration was 3% (mass fraction), the polishing speed was 80 r/min, and the polishing pressure was 20 kg. After the Si polishing of the 4H-SiC wafer for 60 min using this set of parameters, the surface roughness R_a_ decreased from 114.8 to 0.9 nm, with a change rate of 99.2%. After further polishing for 60 min, the ultrasmooth surface with a surface roughness R_a_ of 0.5 nm and an MRR of 20.83 nm/min was obtained;
3. The shear rheological polishing method can effectively remove scratches on the Si surface of 4H-SiC wafers and improve the Si surface quality of 4H-SiC wafers. This study provides a new method for polishing the Si surface of 4H-SiC wafers with high efficiency and quality.

## Figures and Tables

**Table 1:** SiC wafer material properties.

Parameter	Value
Crystal type	4H
SiC content (%)	99.9
Density (g/cm^3^)	3.12
Mohs hardness	9.2
Poisson ratio	0.18

**Table 2:** SRP parameters.

Parameter	Value
Workpiece speed (r·min^−1^)	20
Polishing speed (r·min^−1^)	70, 80, 90
Pressure (kPa)	9.05, 10.19, 11.32
Concentration (wt%) of diamond particles	3, 6, 9
Size of diamond particles (μm)	0.5, 1, 1.5
Experimental observation interval (min)	30
Polishing fluid flow rate (ml·min^−1^)	12

**Table 3:** L9(3^4^) Orthogonal experimental.

i	Combination	Abrasive Particle Size (A) (μm)	Abrasive Concentration (B) (wt%)	Polishing Speed (C) (r·min^−1^)	Pressure (D) (kPa)
1	A1B1C1D1	0.5	3	70	9.05
2	A1B2C2D2	0.5	6	80	10.19
3	A1B3C3D3	0.5	9	90	11.32
4	A2B1C2D3	1.0	3	80	11.32
5	A2B2C3D1	1.0	6	90	9.05
6	A2B3C1D2	1.0	9	70	10.19
7	A3B1C3D2	1.5	3	90	10.19
8	A3B2C1D3	1.5	6	70	11.32
9	A3B3C2D1	1.5	9	80	9.05

**Table 4:** Polishing test results and *S*/*N* values.

i	Surface Roughness, R_a_ (nm)						*S*/*N* (dB)	
	Point 1	Point 2	Point 3	Point 4	Average Mean	MRR nm/min	R_a_	MRR
1	59.5	43.6	54.7	41.8	49.9	6.5	−34.06	17.38
2	31.8	50.2	48.3	44.1	43.6	7.8	−32.91	19.82
3	38.4	29.3	35.2	32.3	33.8	13.3	−30.62	22.48
4	9.7	8.3	10.6	7.7	9.1	43.4	−20.86	32.75
5	21.2	27.8	19.7	28.5	24.3	34.7	−26.63	31.93
6	15.3	11.5	11.0	20.2	14.5	30.3	−23.50	29.63
7	12.4	7.4	9.6	8.4	9.5	22.4	−19.68	27.00
8	13.0	15.4	8.9	16.2	13.4	21.9	−22.72	27.92
9	16.7	12.2	15.6	12.5	14.3	28.7	−24.17	29.16

## Data Availability

Data are only available upon request due to restrictions regarding, e.g., privacy and ethics. The data presented in this study are available from the corresponding author upon request. The data are not publicly available due to their relation to other ongoing research.
